# No relations between executive functions and dimensional models of psychopathology or is time the missing link?

**DOI:** 10.1371/journal.pone.0288386

**Published:** 2024-03-11

**Authors:** Hanneke M. E. Feijs, Loes van Aken, William M. van der Veld, Paul T. van der Heijden, Jos I. M. Egger

**Affiliations:** 1 Center of Excellence for Neuropsychiatry, Vincent van Gogh Institute for Psychiatry, Venray, The Netherlands; 2 Donders Institute for Brain, Cognition and Behaviour, Radboud University, Nijmegen, The Netherlands; 3 Behavioural Science Institute, Radboud University, Nijmegen, The Netherlands; 4 Center for Adolescent Psychiatry, Reinier van Arkel Mental Health Institute, ‘s-Hertogenbosch, The Netherlands; 5 Stevig Specialized and Forensic Care for People with Intellectual Disabilities, Dichterbij, Oostrum, The Netherlands; Medical University of Vienna, AUSTRIA

## Abstract

Impaired executive functions (EF) have been found within various mental disorders (e.g., attention deficit hyperactivity disorder, autism spectrum disorder, schizophrenia spectrum disorders) as described in DSM-5. However, although impaired EF has been observed within several categories of mental disorders, empirical research on direct relations between EF and broader dimension of psychopathology is still scarce. Therefore, in the current investigation we examined relations between three EF performance tasks and self-reported dimensions of psychopathology (i.e., the internalizing, externalizing, and thought disorder spectra) in a combined dataset of patients with a broad range of mental disorders (N = 440). Despite previously reported results that indicate impaired EF in several categories of mental disorders, in this study no direct relations were found between EF performance tasks and self-reported broader dimensions of psychopathology. These results indicate that relations between EF and psychopathology could be more complex and non-linear in nature. We evaluate the need for integration of EF and dimensional models of psychopathology and reflect on EF as a possible transdiagnostic factor of psychopathology.

## Introduction

Executive functioning (EF) is defined as a set of cognitive skills that are responsible for planning, initiating, sequencing and monitoring complex goal-directed behaviors and control of complex activities [1–4]. EF is the ability to play with ideas, ‘taking the time to think before acting; meeting novel, unanticipated challenges; resisting temptations; and staying focused’ [5]. According to Lezak and colleagues [6] EF is vital to human autonomy as it involves ‘capacities that enable a person to engage successfully in independent, purposive, self-serving behavior’. As EF influences self-regulation in daily life by reducing problem-behavior and raising more goal-directed behavior, more effective EF led to a more adaptive and successful life [7], whereas executive *dys*function can aggravate negative feelings and behavioral outbursts by the perceived lack of control in life [7, 8]. The commonly used model of EF in scientific literature is the division into three core subfunctions: (1) updating working memory, (2) shifting between task sets, and (3) inhibiting prepotent thoughts and responses [2, 5, 9]. Shifting, updating and inhibition are all needed to some extent when making daily life decisions [7]. Neuroimaging studies have provided further evidence of the multifaceted nature of EF, revealing distinct associations between specific domains of EF and corresponding brain regions [10, 11]. Neuronal and behavioral research suggests that EF skills vary along a continuum from “cool EF” to “hot EF”, suggesting that EF may operate differently in different contexts [12–16]. Cognitive flexibility, inhibition, working memory and more complex EF such as planning and organization are considered aspects of “cool EF”. Emotion regulation, i.e., modulating approach-avoidance reactions, delaying gratification or other affective decision-making, and goal- and future-oriented cognitive processes in contexts that generate motivation, are considered “hot” affective aspects of EF [17].

Impairments in EF can be considered a shared underlying key component in a broad range of mental disorders [18–20]. Nonetheless, empirical findings from studies investigating the associations between EF and mental disorders in adults are inconsistent [21]. Impairments in EF have been found in various categories of mental disorders, as categorized by the Diagnostic and statistical manual of mental disorders (DSM) [22] such as eating disorders [23, 24], anxiety disorders [25], obsessive-compulsive disorder (OCD; [26], depression [27], schizophrenia spectrum disorders [28, 29], psychotic symptoms in at-risk youth [30], autism spectrum disorders (ASD; [31, 32], attention-deficit/hyperactivity disorder (ADHD; [33], conduct disorder [34], antisocial behavior [35, 36], borderline personality disorder [37], learning difficulties [38], and fetal alcohol spectral disorder [39]. However, it is important to note that these impaired EF findings were studied indirectly within categories of mental disorders and are not in direct relation to psychopathology. Also, researchers argue that the assumptions of the categorical approach to psychopathology is flawed, and that psychopathology should be considered as dimensional [40].

As EF is consistently found to be impaired in various disorders, in recent years, EF is considered a transdiagnostic indicator of psychopathology [41–43]. The relation between impairments in EF and a tendency to gradually develop psychopathology later in life has been demonstrated by Martel et al. [44] in a cross-sectional study of 2,395 children (aged 6–12 years). Martel and colleagues found that the performance on EF measures was associated with risk for a general psychopathology factor (but not for specific factors). In line with these findings, Caspi et al. [45] found that several cognitive and behavioral EF measures demonstrated meaningful negative associations with general psychopathology (see table p.129). These findings have led to the hypothesis of a typical dysfunctional developmental pathway that leads from adverse childhood experiences (ACEs) and other stressors to the disruption of a healthy development of neural systems. As these neural systems are known to underly and support EF, disruptions could lead to psychopathology and, in turn, lead to more stress which undermines EF even further [42, 46–48]. Furthermore, researchers are pointing towards evidence of a common genetic ’background’ for EF and psychopathology [49–51]. Overall, these findings of different studies support the hypothesis of a typical (dys)functional developmental pathway and the understanding of shared etiological influences between cognitive functioning and mental health.

Impairments in EF have also been studied in non-clinical adult samples. Roye et al. [19] conducted one of the few studies in the field studying direct relations between different domains of EF and psychopathology in a non-clinical adult sample. The study conducted by Roye et al. [19] examined relationships between individual domains of EF, using multiple subtests (Delis-Kaplan Executive Function System, Adult Self-Report, Peter’s et al. Delusions Inventory), while accounting for non-EF factors. The results with confirmatory factor analyses and comparing models with structural equation modeling revealed both positive and negative associations between internalizing and externalizing symptoms and EF domains, as well as differences in EF performance among the three psychopathology factors. More specifically, more severe internalizing symptoms were associated with reduced fluency performance but improved shifting and inhibition task performance. Conversely, externalizing symptoms were linked to poorer inhibition task performance but better fluency task performance. Individuals with greater internalizing symptoms exhibited enhanced performance on both inhibition and shifting tasks. It is worth noting that timed measures, which include a speed-accuracy tradeoff, can influence the interpretation of EF and psychopathology relationships. For instance, increased anxiety may not affect inhibition or shifting accuracy but can result in slower performance. The study also revealed a negative association between internalizing symptoms and fluency performance, aligning with previous findings suggesting a negative correlation between worry, rumination, and performance on updating tasks. In contrast, individuals with greater externalizing pathology exhibited poorer inhibition task performance and better fluency task performance. In conclusion, the authors describe the importance of considering task impurity in EF measures, particularly regarding processing speed, and highlight the influence of non-EF variables when interpreting relationships between EF, psychopathology, and objective performance measures.

Despite the observed impairment of EF in various mental disorder categories, empirical research on direct relationships between EF and broader dimensions of psychopathology, and their interactions, remains limited. Therefore, in this study, we analyzed direct relation between EF and broader domains of psychopathology (i.e., internalizing, externalizing and thought disorder) in a clinical sample of adults. By focusing on a clinical adult sample, we hope to provide valuable insights into the specific relationships between EF and psychopathology in a population that often exhibits more severe symptomatology and functional impairment compared to younger community samples. It is expected that relations with EF will be found with each of these dimensions of psychopathology. The results will be discussed in detail in the subsequent sections.

## Method

### Participants and procedure

Participants in this study were patients from the Vincent van Gogh Center of Excellence for Neuropsychiatry in Venray, Netherlands, a neuropsychiatric department of a Dutch psychiatric hospital. This is a specialized center for patients who had a history of (partially) unsuccessful mental health care for on average 5–7 years before admission. Patients are sent to the center for an in-depth diagnostic evaluation. Typically, before the referral, multiple comorbid DSM-IV and DSM-5 diagnoses were previously established. For example, patients were diagnosed with major affective (including bipolar) disorder, anxiety disorders, impulsivity related psychopathology, developmental disorders, personality disorders and, to a lesser extent, psychotic disorders. Also, these diagnoses are often paralleled by one or more of the following: a history of school failure, adverse childhood experiences, cognitive complaints, difficulties with emotion regulation and social cognition, or multiple somatic complaints including various genetic syndrome diagnoses. It is important to note that while the center offers treatment for various mental health conditions, patients with forensic problems and addictions are typically treated in separate departments within the organization. The sample in this study consisted of 65% self-identified women and the mean age was 37.60 years (*SD* = 14.17). Full-scale IQ (FSIQ) were measured for 429 participants by the third or fourth edition of the Dutch version of the Wechsler Adult Intelligence Scale [52, 53] and ranged from 82 to 106. For individuals with FSIQ scores below 85 verbal reasoning abilities and comprehension were judged to be sufficient to complete the questionnaire. Also, the test administrator was available to answer questions concerning the items. Moreover, all patients included in this study met the validity cutoffs of the on the Minnesota Multiphasic Personality Inventory–2 Restructured Form (MMPI-2-RF) profiles (i.e., CNS < 14, VRIN-r/TRIN-r < = 80T, F-r < 120T, F-r < 100T, L-r < = 80T), which are standard procedure in the psychological assessment. Education level, according to the Dutch educational system and ranging from category 1 (less than six years of primary education) to 7 (academic degree; [54]) were available for 253 participants and varied from 2 to 7. It is common that patients in this center receive medication. Cognitive assessments are, however, not conducted during changes or adjustments in pharmacological treatment. Data-collection was part of standard assessment procedures that include extensive intellectual, neuropsychological and personality assessment, and usually covers three three-hour sessions within two weeks.

All patients have been given their informed consent to process their data anonymously in a database for scientific research. Anonymous patient records for the current study were drawn from this large electronic database, containing patients’ test results from March 2005 to September 2018. Patients were included in this study if, in the database, they had valid scores (i.e., other values than 1 or 2) on the MMPI-2-RF questionnaire, valid EF scores (i.e., no impossible scores), and scores on at least two of the three EF tasks (that were analyzed in this study). Patients were excluded from the study when they had an intellectual disability, as this may affect cognitive functioning and confound the results, when they were in a current episode of acute psychiatric symptoms, such as severe depression, mania, or psychosis, as this may affect both neuropsychological functioning and self-reported psychopathology measures. Patients were also excluded from the study when they were not fluent in the Dutch language, as this may affect their ability to complete the cognitive evaluation and self-report measures accurately, and when they were not able to provide informed consent to participate in the study. In total 440 in- and outpatients met these inclusion and exclusion criteria. For four participants demographic information was missing and for 17 participants the precise adult age was unknown.

### Measures

#### Measures of EF

EF tests were chosen as they are commonly used traditional EF performance tasks in regular neuropsychological assessment procedures. It is noteworthy to mention that the chosen tests of measuring EF do not fully cover the beforementioned traditionally used model of EF in scientific literature, which divides EF into three core subfunctions (i.e., updating working memory, shifting between task sets, and inhibiting prepotent thoughts and responses; [2, 5, 9]).

*Stroop test (n = 428)*. The Stroop Colour-Word test [55] is one of the most widely used tests in neuropsychology [55] and can be considered a test that assesses mental speed, executive attention, and response inhibition. During the administration of the Stroop test, participants are instructed to read colors aloud on three cards, successively, as fast as possible. On the first card (the word-card) participants are asked to read color names in black ink, on the second card (the color-card) participants read color squares that are printed in a random order, and on the third card (the color-word-card) participants are asked to read only the, incongruent, color in which the color names are printed (i.e., the word ‘yellow’ is printed in red, blue or green ink alternately), hence creating an interference effect. For the test, reported reliability coefficients range from moderate to good [55]. For the current analyses in this study, the interference score was used to measure response inhibition, which is obtained by dividing response time on the third card by the average response time of the first and second card. Higher scores indicate more interference, therefore poorer performance.

*Trail Making Test (n = 399)*. The Trail Making Test (TMT; [56]) is a widely used test in clinical practice and is considered sensitive to cognitive impairment associated with a wide variety of neurological disorders [6, 57, 58]. The TMT aims to investigate divided attention and cognitive flexibility by measuring the speed of information processing, visual search behavior and visual motor skills. It consists of two tasks: on the first task (part A) 26 encircled numbers that are randomly distributed on a sheet of paper must be connected in an ascending order. In the second task (part B), numbers (1–13) and letters (A-L) must be connected alternately and in ascending order (i.e., 1, A, 2, B, 3…). The patient is instructed to perform the task as quickly and accurately as possible, while not letting go of the pencil. Part A is considered to measure basic abilities such as motor speed and visual search, whereas part B examines mental flexibility. The reported reliability coefficients for the TMT time scores range from moderate to good (see [55]). For analyses in this study, the ratio score of completion times of card B divided by completion times of card A were included. A higher score indicates poorer performance.

*Tower of London (n = 314)*. The Tower of London (ToL) test is a neuropsychological instrument designed to assess mental planning ability [59], which is an important domain in EF. During the administration of the ToL test, subjects are given a wooden board with three posts onto which colored beads (in red, green, and blue) must be arranged. The respondent is asked to get from a fixed starting position of the beads to a target position, by moving the beads between the three posts. It is emphasized that the end position must be reached in as few movements as possible, without breaking provided rules. The reported reliability coefficients range from moderate to good (see [55]). For analyses in this study, we used the total number of steps (bead moves) required to get to the target position. A lower score indicates a poorer performance.

#### Dimensional measures of psychopathology

*The MMPI-2-RF (n = 440)*. The Dutch-language version of the Minnesota Multiphasic Personality Inventory–2 Restructured Form (MMPI–2–RF; [60, 61]) is a self-report questionnaire assessing personality traits and dimensions of psychopathology. The MMPI-2-RF consists of 338 items, eight validity scales, three higher-order (H-O) scales, 10 restructured clinical scales, 23 scales targeting specific psychopathological symptoms, two interest scales, and five personality psychopathology scales (revised; PSY-5-r). Protocol validity is measured by judging inconsistent responding; fixed responding; overreporting of psychological, somatic, or cognitive symptoms; and underreporting response styles. The three higher-order scales are Emotional-Internalizing Dysfunction (EID), Thought Dysfunction (THD), and Behavioral-Externalizing Dysfunction (BXD). They consist of 41 items, 23 items and 26 items respectively. Under the EID scale, RCd (Demoralization) and RC7 (Dysfunctional Negative Emotions) subscales assess feelings of hopelessness, worthlessness, despair, and symptoms of depression, such as sadness, guilt, and lack of pleasure, whereas RC2 (Low Positive Emotions) evaluates an individual’s inability to experience positive emotions. For the EID scale, T-scores between 65 and 79 indicate increased levels of emotional distress. A higher T-score of 80 on the EID scale suggests significant emotional turmoil, often associated with experiencing a crisis. Under the THD scale, RC6 (Ideas of Persecution) and RC8 (Aberrant Experiences) subscales measure cognitive and perceptual disturbances, such as paranoid ideation, suspiciousness, unusual experiences, and difficulties with concentration, memory, and decision-making. T-scores ranging from 65 to 69 on the THD scale suggest clear disturbances in thinking, while a T-score of 80 indicates a serious disturbance in cognitive functioning. Under the BXD scale, RC4 (Antisocial Behavior) and RC9 (Hypomanic Activation) subscales assess rule-breaking behavior, criminality, disregard for others, and impulsive, risk-taking behavior, respectively. Regarding the BXD scale, T-scores between 65 and 69 indicate answers that clearly indicate externalizing and/or acting-out (disinhibited) behavior, which may lead to trouble or difficulties. A T-score of 80 suggests significant externalizing and/or acting-out behavior that is very likely to result in marked dysfunction and problems. Detailed information about the psychometric properties of the Dutch-language version of the MMPI-2-RF in the Dutch normative sample and clinical samples is provided by Van der Heijden, Egger, and Derksen [62, 63]. Scores were computed from administration of the MMPI-2-RF booklet (n = 76; 17%) and administration from the MMPI-2 booklet (n = 364; 83%). Ben-Porath and Tellegen [60], and Van der Heijden et al. [63], confirmed comparability of scores derived from both booklets. The three higher-order scales index broadband psychopathology constructs of, respectively, internalizing, thought disorder, and externalizing symptoms. They map onto broader level dimensions of the Hierarchical Taxonomy of Psychopathology model (HiTOP; see, [40, 64, 65]) and are therefore included for current analyses with raw scores as dimensional measures of psychopathology. It is noticeable to highlight that the time frame of the three higher-order MMPI-2-RF scales differs across scales, with the externalizing disorders scale encompassing a longer period, including items about early childhood experiences. For example, items related to aggression and behavioral problems may reflect patterns observed over a longer duration. We have included this information in the Methods section of this study to provide clarity on the temporal focus of the assessment.

### Statistical analyses

**Fig 1:**
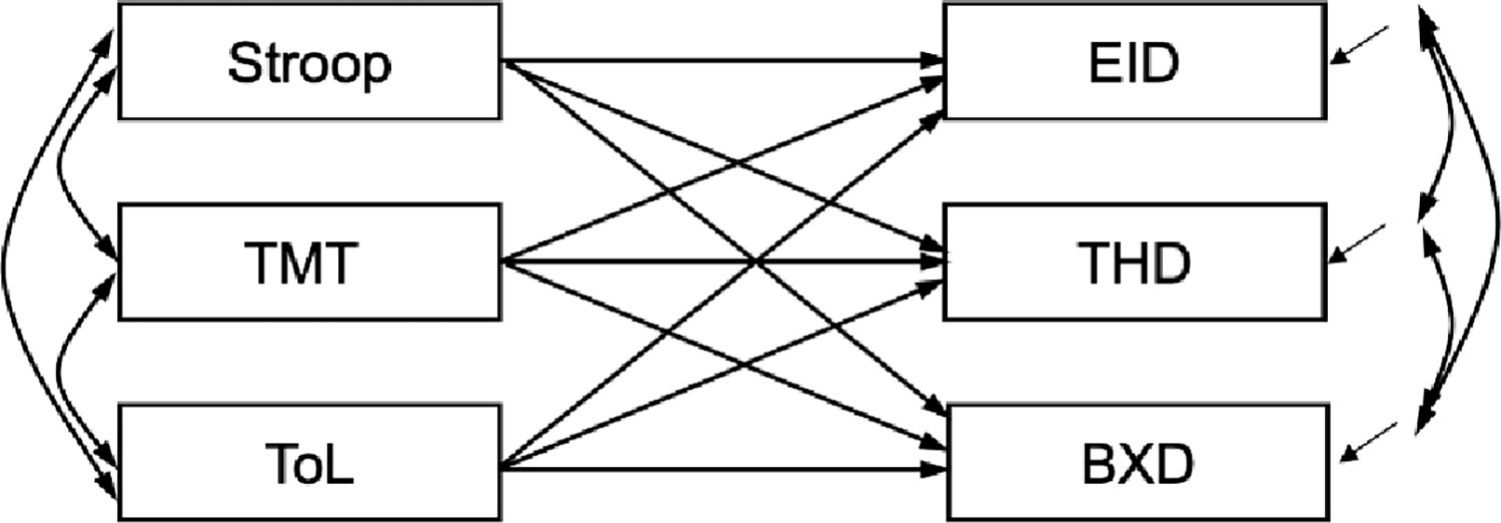
Multivariate regression model to simultaneously study the relations between executive functioning and psychopathology. Note: Stroop = Stroop test, TMT = Trail Making Test, ToL = Tower of London test, EID = Emotional/Internalizing Dysfunction, BXD = Behavioral/External Dysfunction, THD = Thought Dysfunction.

Descriptive statistics and correlations were obtained using IBM SPSS Statistics version 27 (released 2020; [66]). To study the relationship between executive functioning and psychopathology we analyzed the multivariate regression model (Fig 1). This model allows simultaneous estimation of the relations between the EF measures (Stroop, TMT, ToL) in our study and psychopathology (EID, BXD, THD) as measured with the MMPI2-RF. For the analyses, the scores of the MMPI-2-RF scales were transformed in non-gendered T scores, following the instructions in the manual [60]. We used lavaan [67], an R-package, to estimate the model in Fig 1. All model parameters were estimated using the Maximum Likelihood (ML) estimator. Table 1 shows that the observed scores of the Stroop and the TMT are not normally distributed, that is, kurtosis and skewness indicate strong deviations. To account for these deviations from normality, we have estimated robust standard errors. There were no missing scores on the EID, BXD, THD, but the Stroop, TMT, and Tol did have missing scores, respectively 2.7% (n = 12), 9.3% (n = 41), and 28.6% (n = 126). In order to use all available scores in the estimation we used the full information variant of the ML estimator.

**Table 1:** Descriptive statistics of the EF measures and the MMPI-2-RF scales (N = 440).

	*M*	*SD*	Range		Skewness	Kurtosis
			Min.	Max.		
Stroop	2.05	.52	.18	5.72	1.87	8.86
TMT	1.72	.75	.72	6.07	2.23	7.16
ToL	26.19	3.97	16.00	35.0	-.18	-.36
EID	67.73	13.16	36.00	90.00	-.39	-.70
BXD	58.95	16.56	34.00	100.00	.64	-.23
THD	59.59	15.64	38.00	100.00	.85	.24

*Note*. Stroop = Stroop test, TMT = Trail Making Test, ToL = Tower of London test, EID = Emotional/Internalizing Dysfunction, BXD = Behavioral/External Dysfunction, THD = Thought Dysfunction.

## Results

All participants completed the MMPI-2-RF questionnaire. Descriptive statistics with normed scores presented that a significant proportion of the sample exhibited elevated levels of emotional distress. Specifically, 57.7% of the participants scored above the cut-off score on the Emotional/Internalizing Dysfunction (EID) scale, indicating increased levels of emotional distress. Furthermore, 18.6% of the participants scored above the more stringent cut-off score on the EID scale, suggesting significant emotional turmoil. In terms of thought disorders, a subset of the sample displayed indications of cognitive dysfunction. Approximately 22.7% of the participants scored above the cut-off score on the Thought Dysfunction (THD) scale, implying a clear disturbance in thinking. Additionally, 8.6% of the participants surpassed the higher cut-off score, indicating a more serious disturbance in thinking. Regarding behavioral problems and under-controlled or impulsive behavior, a notable proportion of the sample exhibited signs of externalizing and acting out behaviors. Specifically, 25.5% of the participants scored above the cut-off score on the Behavioral Problems (BXD) scale, suggesting the presence of externalizing and/or disinhibited behavior that may lead to difficulties. Moreover, 11.1% of the participants indicated significant externalizing and acting-out behavior, which is likely to result in marked dysfunction and problems. Overall, these findings demonstrate that the sample consisted of individuals with diverse psychopathological symptoms. In sum, participants reported most often emotional distress, followed by thought disturbances and behavioral problems in nearly equal manner.

Descriptive statistics with raw scores for EF measures and psychopathology measures are presented in Table 1. Table 2 presents the Pearson correlations between EF and the MMPI-2-RF scales. The correlations among the EF measures were rather weak, between *r* = -.14 (*p* = .023) and *r* = .18 (*p* = .003). The directions of the correlations were in line with the scoring of the measures. The correlations among the higher-order dimension of the MMPI-2-RF are somewhat stronger. The correlations vary between *r* = -.17 (*p* = .007) and *r* = .38 (*p* < .001), this is to be expected as they originate from the same scale.

**Table 2:** Pearson correlations between EF and MMPI-2-RF scales (N_listwise_ = 261).

	Stroop	TMT	ToL	EID	BXD	THD
Stroop	1					
TMT	.18**	1				
ToL	-.14*	-.18**	1			
EID	.01	-.06	.00	1		
BXD	-.01	.01	-.11	.17**	1	
THD	.06	.05	-.10	.35**	.38**	1

Note

** *p* < .01

* *p* < .05; Stroop = Stroop test, TMT = Trail Making Test, ToL = Tower of London test, EID = Emotional/Internalizing Dysfunction, BXD = Behavioral/External Dysfunction, THD = Thought Dysfunction.

### Multivariate regression analyses

The results of the analysis of the multivariate regression model (Fig 1) are presented in Table 3. The fifth column contains the r-squared for the psychopathology measures. The numbers indicate that the explained variance varies between 1% and 2%. In addition, all the estimated standardized regression weights were nonsignificant, meaning that there was no relation between the EF measures and psychopathology measures. In hindsight, the correlations between all the measures (see Table 2) painted the same picture.

**Table 3:** Standardized regression weights (β) in the multivariate regression model (N = 440).

	Stroop	TMT	ToL	R^2^
EID	.05	-.05	-.02	.01
BXD	-.00	-.03	-.08	.01
THD	.05	.04	-.09	.02

Note

** p < .01

* p < .05

Stroop = Stroop test, TMT = Trail Making Test, ToL = Tower of London test, EID = Emotional/Internalizing Dysfunction, BXD = Behavioral/External Dysfunction, THD = Thought Dysfunction.

## Discussion

In this study, relations between EF and three main spectra of the psychopathology dimensions (i.e., internalizing, externalizing and thought disorder) were studied. No correlations between EF tasks and (self-reported) psychopathology measures were found in the current sample. This is contrary to results from a body of literature reporting consistently evident (indirect) links between EF and psychopathology. We will discuss several considerations, among which the type of measuring methods (performance tasks versus self-report measures), the definition and measurement of EF, and the practical value of the EF concept for both clinical practice and empirical and future research.

Prior to the more detailed discussion, it is worth noting that significant relations between executive functioning (EF) and dimensional measures of psychopathology were expected in this study, given the clear presence of psychopathological symptoms in the sample and prior evidence of EF impairment across various categorical disorders. Regarding emotional/internalizing dysfunction (EID), more than half of the sample (i.e., 58%) scored above the cut-off score (T ≥ 65), indicating increased levels of emotional distress. Furthermore, a notable percentage (19%) surpassed the higher cut-off score (i.e., T = 80), suggesting the presence of significant emotional turmoil. These results highlight the considerable emotional burden experienced by patients in this sample. In terms of the thought disorder scale (THD), a considerable proportion of the participants (23%) scored above the cut-off score (T ≥ 65), indicating clear disturbances in thinking such as paranoid and non-paranoid delusions, auditory or visual hallucinations, unrealistic thinking. Moreover, 9% of the participants surpassed the higher cut-off score (i.e., T = 80), pointing to more serious disturbances in thought functioning. These findings suggest that a significant portion of the sample experiences significant levels of psychopathology, that may impact their daily functioning and overall well-being. Also, regarding behavioral problems, specifically problems with under-controlled behavior (BXD), a substantial percentage of the sample (26%) scored above the cut-off score, indicating the presence of externalizing and/or acting-out behaviors. Additionally, 11% of the participants surpassed the higher cut-off score (i.e., T = 80) on the BXD scale, indicating significant externalizing and acting-out behavior. These elevated scores reflect the complexity and severity of psychopathology present in the sample. Considering these findings, it is noteworthy that no significant relations between EF and dimensional measures of psychopathology were found when directly studied using sophisticated statistical analyses.

First, we will discuss three key factors that could contribute to the absence of significant correlations in this study: the different measuring methods used, the difference in timeframes of action between the measures, and the potential misalignment between dimensions of psychopathology and momentary fluctuations in cognitive performance. Regarding the associations between performance tasks of EF and self-report measures of psychopathology, it has been suggested that larger sample sizes and more sophisticated statistical modeling methods, such as structural equation modeling, are necessary to better clarify neuropsychological functioning relations to report-based trait or symptom measures [68]. However, relations between related constructs assessed using different measuring methods can be expected to correlate only modestly and effect sizes can be expected to be relatively small [69]. Furthermore, and more importantly, low correlations and low effect sizes could also be logical since the measurement contexts for our self-report and performance tasks in neuropsychological and psychopathology measures differ in their timeframe of action (i.e., life-long timeframe vs current moment ‘snapshot’; [70, 71]). Considering this, it could be reasonable to expect that higher order dimensions of psychopathology that may have trait-like characteristic may not strongly align with momentary fluctuations in cognitive performance. As no correlations were found in the currents study, even though a large clinical sample size and solid statistical analyses were used, the difference in these measurement contexts might contribute to the explanation for the absence of correlations.

Additionally, it is, overall, noticeable that the sole reliance on self-report measures is common in psychopathology research [72–76], and relatively few studies assess the external validity of factorially derived constructs using alternative measures and methods (i.e., performance-based tests). Dimensional models of psychopathology mostly draw on findings from factor-analytic investigations of self-report measures, collected using diagnostic interview and questionnaires, and not based on a multimethod approach. However, introspective limitations constrain the accuracy of self-reports [77, 78]. Not surprisingly, self-reports diverge from reports provided by knowledgeable informants [79, 80], and correlations between self-reported behavior and actual behavior exhibited in laboratory, clinical, and field settings are modest, typically in the range of .2 to .3 [81, 82]. Regarding these limitations it is therefore strongly advised to study relations between psycho(patho)logical and neuropsychological functioning by means of a multimethod and multi-informed approach that integrates, for example, both self-report measures and performance tasks [74, 83].

Secondly, the definition and measurement of the neuropsychological concept EF will be discussed. Starting at the traditional definition of EF, the widely used and adopted three-factor classification includes updating working memory, shifting between task sets, and inhibiting prepotent thoughts and responses [2, 5, 9]. However, the model and construct ‘EF’ is subject to ambiguous conceptualization and operationalization difficulties. This is reflected in the current low intercorrelations between the EF tasks, which can imply that they are measuring different aspects of EF and could also reflect the diffuse operationalization of the EF construct. Recent research has suggested a hierarchical bifactor model involving a ‘common EF’ latent variable together with updating (working memory) and shifting (cognitive flexibility) variables [10]. The authors declare that this unity/diversity framework may be a better fit to the different EF processes [84, 85]. The note that even when discussions on the definition and conceptualizations of EF are cleared up, traditional tests of EF are then still subject to the problem of task-impurity. The task impurity problem in the assessment of EF points to the problem that it is often unclear exactly which areas of EF are being measured by which measurement task, and how much each task assesses multiple constructs of EF. It is plausible that many EF tasks could involve more than one executive process, making it difficult to measure or understand isolated areas of EF in depth, and furthermore, leading again to little consensus about the underlying processes of EF. Therefore, the problem of task impurity could have influenced current findings, although it is unknown to what extent. Furthermore, performances on lab and questionnaire assessments of EF do not relate consistently [86, 87]. However, overall, it has been shown that standard performance tasks predict independent variance when compared with questionnaires [86, 88]. Within the current study, only performance tasks of EF were measured, and it should be considered that the additional usage of EF self-report questionnaires could possibly have led to different conclusions. Further research could study this possibility in more detail.

Moreover, considering the practical value of the EF concept for research purposes, operationalization problems will be discussed in this next section. Within the literature, studies can be found reporting strong links between EF and psychopathology, whereas other studies, as well as the current analyses, do not find any significant relations. This is due to the very broad and vague operationalization of the EF concept, which has been elaborated on in more detail in the previous sections. Additionally, it should be noted that heterogeneous findings in research of neuropsychological (EF) and psychopathology research are mostly due to the reliance on the DSM classifications [89], where EF is indirectly measured in various ways within the different categories. As it is valuable to do so, based on a large body of scientific literature, we adopted EF as an important transdiagnostic factor in psychopathology. We conclude that the lack of cohesion between both EF measures and self-report questionnaires of psychopathology, and between lab and questionnaire assessments in EF, strongly pleads for the use of a multi-method dimensional approach not only in scientific research, but also in clinical practice, to optimally capture the breadth of EF. Researchers and clinicians should be informed to adopt this approach as a better fit of operationalizing EF, to bridge the gap in neuropsychological and psychopathological research, as more research is needed to understand these relations.

Additionally, regarding the definition and variability of EF skills based on emotional context, and its relevance in studying the relations between neuropsychological and psychopathological functioning, a body of literature discusses that EF skills are not stable in time, but rather vary with emotional status along a continuum from “cool EF” to “hot EF” [12–16]. Here, “cool EF” refers to EF skills assessed in relatively emotionally neutral contexts such as working memory, planning and organization, and “hot EF” refers to EF skills that are needed in situations that are motivationally (e.g., affectively) significant such as self-control, ability to (not) procrastinate, deliberate emotion regulation and social cognition, often involving an incentive or reward component. This differentiation of EF is expected to be very relevant in the study of relations of neuropsychological and psychopathological functioning since human beings do function differently over time and are very much influenced by their emotions. Consideration of hot and cool EF has helped characterize a wide range of clinical conditions [48] including ADHD [33, 90], ASD [32], antisocial personality disorder [35], conduct disorder [34], developmental coordination disorder [91], fetal alcohol syndrome disorders [39], OCD [92], sequelae of prematurity [93], psychotic symptoms in at-risk youth [30], and the consequences of traumatic brain injury [13]. However, also failures to find differences between hot and cool EF across clinical conditions have been reported [94]. We want to point out that all EF measures within this study are considered cool EF skills [48], as traditional EF measures generally are. Considering the growing appreciation of the role of hot EF and motivational difficulties such as delay aversion, temporal discounting, and emotion dysregulation [33] in psychopathology research, using additional hot EF measures in our study could have also led to different results and therefore sharpen our understanding of EF relations with psychopathology.

One important finding and limitation of our study is the disparity in findings compared to the study by Roye et al. [11], which demonstrated direct relationships between EF and psychopathology in a non-clinical, open-access sample with limited psychiatric exclusion criteria focused on severe mental illness. The differences in sample characteristics, specifically the inclusion of individuals with severe mental illness in our study, as well as the variations in diagnostic profiles and exclusion criteria, may account for the discrepancy in findings. Moreover, it is important to note that our study employed SEM analyses while the study by Roye, Calamia and Robinson employed confirmatory factor analyses. These methodological differences may have also influenced the contrasting results. Future research could further investigate the impact of sample characteristics and statistical analysis methods on the relationship between EF and psychopathology in both clinical and non-clinical populations to gain a more comprehensive understanding of these associations (see for more the recommendations for future research in the conclusions paragraph).

In sum, a limitation of this study is that the sample group consisted of patients who had been in psychiatric care for a long time. As such, the findings from this study may not be generalizable to all patients, particularly those who have not received long-term care. Additionally, the sample included individuals with a range of psychiatric symptom severity, and diagnostic frequencies for the current sample become increasingly important, as individuals with severe mental illness, particularly those diagnosed with a psychotic spectrum disorder, commonly exhibit lower baseline functioning in neuropsychological assessments. Another limitation is that medication information could not be analyzed in this study. This poses a limitation to the study as treatment with antipsychotics or opioids can impact aspects of cognition, such as processing speed, which is embedded within two of the three cognitive measures used in the current study. However, given the heterogeneity of the group, these effects are likely to be diffuse, as the group is not characterized by one type of medication. Moreover, no testing was done during pharmacological adjustments, although medication effects cannot be excluded. Furthermore, confounding factors, such as age, gender, educational level, socioeconomic status, and pre-morbid intelligence, were not explicitly considered in the statistical analyses of this study or discussed as limitations in the relations between EF and dimensional measures of psychopathology.

## Conclusions

In conclusion, our findings state that significant relations between EF and dimensional measures of psychopathology could not be found in a relatively large heterogeneous group of adult psychiatric patients, although a large sample size and a sophisticated statistical modeling method (structural equation modeling) was used. We believe that these results can be reproduced with similar EF measures and self- or informant-report questionnaires in clinical and non-clinical samples due to the different measuring methods. However, we caution that performance measures and self-reported data may not fully capture the dynamic processes of symptomatology, where symptoms are often causally interrelated to other distinct processes [95]. For future research, it may be beneficial to consider incorporating clinician-rated rating scales alongside self-report measures. Additionally, to overcome the problem of the different measurement contexts (self-report versus performance measures), research that uses experience sampling methods and frequent assessment of behaviors, emotions, and thoughts might be particularly helpful in capturing the time course of these symptomatic relationships between EF and psychopathology [44, 96]. These more dynamic measures of both the cognitive domain (event related cognition), psychopathology (i.e., behavioral analysis) and fitting dynamical statistical analysis (i.e., time-series based analysis) could possibly do more justice to the dynamic processes of symptomatology and human behavior in general, and we therefore recommend these analyzing techniques for future research on EF and psychopathology.

## Supporting information

S1 Data (SAV)
